# Cascading Failure Analysis on Shanghai Metro Networks: An Improved Coupled Map Lattices Model Based on Graph Attention Networks

**DOI:** 10.3390/ijerph19010204

**Published:** 2021-12-25

**Authors:** Haonan Ye, Xiao Luo

**Affiliations:** 1 Urban Mobility Institute, Tongji University, Shanghai 200092, China; haonanye@outlook.com; 2 School of Transportation Engineering, Tongji University, Shanghai 200092, China

**Keywords:** cascading failure, metro network, coupled map lattices, graph attention network

## Abstract

Analysis of the robustness and vulnerability of metro networks has great implications for public transport planning and emergency management, particularly considering passengers’ dynamic behaviors. This paper presents an improved coupled map lattices (CMLs) model based on graph attention networks (GAT) to study the cascading failure process of metro networks. The proposed model is applied to the Shanghai metro network using the automated fare collection (AFC) data, and the passengers’ dynamic behaviors are simulated by GAT. The quantitative cascading failure analysis shows that Shanghai metro network is robust to random attacks, but fragile to intentional attacks. Moreover, there is an approximately normal distribution between instant cascading failure speed and time step and the perturbation in a station which leads to steady state is approximately a constant. The result shows that a station surrounded by other densely distributed stations can trigger cascading failure faster and the cascading failure triggered by low-level accidents will spread in a short time and disappear quickly. This study provides an effective reference for dynamic safety evaluation and emergency management in metro networks.

## 1. Introduction

With the development of the metro network, it has become the main means of transportation in mega-cities. For example, Shanghai has a metro system with 303 stations and 350 tunnels over 617 km [1]. The metro system has great influence on the travel of residents, the efficient use of land and the urban safety, so the research on its security and efficiency has become an important content.

As the metro system plays an increasingly important role in the urban mobility, if the metro system breaks down, the consequences will be extremely serious and unbearable. In the metro system operation, the negative result is usually caused by cascading failure, which can be triggered by the signal failure of infrastructure, natural disasters, terrorist attacks and other emergencies [2]. For example, on 16 April 2018, Shanghai metro system suffered the most simultaneous failures in history, leading to many passengers being stranded. In St. Petersburg, on 3 April 2017, a terrorist attack killed at least 14 people and injured more than 120, which shut down several important metro lines and stranded many passengers [3]. Most of the existing studies on cascading failure based on a static topological network ignore the passengers’ dynamic behaviors between different station pairs. Therefore, in this paper, the passengers’ dynamic behaviors, which are quantified as flow coupling strength between different station pairs, are trained from the Shanghai metro AFC data instead of assuming that all stations have the same flow coupling strength. Moreover, the cascading failure process and robustness optimization of metro networks are studied by an improved coupled map lattices model [4] based on graph attention networks [5] proposed by us. 

This paper provides an advanced and accurate analytical model and theoretical support for the safety operation and the emergency management of metro systems. The main contributions of this study are as follows. (1) An improved CMLs model based on GAT is proposed, which can better reflect the cascading failure of metro networks than the previous coupling models. (2) Based on the coupling strength trained by GAT, the passenger flow redistribution during the cascading failure is dynamically simulated. (3) A case study with the AFC data of the Shanghai metro system demonstrates the topological characteristics of the Shanghai metro, the failure degree under intentional attacks (IAs) and random attacks (RAs) and the influence of the propagation process on the robustness. The rest of this paper is organized as follows. In Section 2, the relevant research is summarized and the research gap is highlighted. Section 3 presents the improved CMLs model based on GAT and the detailed execution procedure. Based on the proposed model, a case study on the Shanghai metro system with Shanghai AFC data is conducted in Section 4. Finally, the conclusions and future research directions are summarized in Section 5.

## 2. Literature Review

### 2.1. Topology Analysis of Metro Networks

In the early stage, the network-based analysis approach [6,7] provides an effective and logical basis to the quantification of the reliability and robustness of large-scale transportation systems. Latora and Marchiori [8] investigated the small-world feature of the Boston metro network based on complex network theory, and then some studies quantified the static topological characteristics of several Chinese metro networks [9,10,11]. The results show that the node connectivity in the small-world networks follows a scale-free power-law distribution.

However, the above network-based studies do not offer a rigorous comparison among metro networks. In this respect, the topological characteristics of the world’s 20 largest metro systems [12] and the complexity and robustness of 33 metro systems in the world [13] were analyzed, which suggest that most metro networks are indeed scale-free networks and small-world networks.

In addition, as there exists the dynamic passenger flow in the metro networks, some studies loaded dynamic passenger flow data into the metro networks based on the static network topologies [14,15,16]. The result suggests that the failure of one or a few nodes could lead to other nodes’ failure by the flow coupling strength between nodes, triggering cascading failure of a few or even all nodes in the network.

### 2.2. Cascading Failure

Some empirical studies on the cascading failures in different networks [17], such as the US power grid and water gird, found that the cascading failure phenomenon had a great impact on the network function. Among the classical proposed models, the load-capacity model started with the work of Kim and Motter [18] has been used to investigate the cascading failure of metro networks. A load-capacity optimal relationship model is proposed and applied to the Shanghai metro network [19]. The results show that the load-capacity optimal relationship model has the best robustness against cascading failure with less cost. Moreover, the cascading failure of the Nanjing metro network [20] is analyzed based on the proposed model considering load fluctuation and robust station capacity assignment. The results show that coupling effects of station capacity on the metro network robustness can be optimized by adjusting model parameters.

Compared with the load-capacity model, the CMLs model [21] has the advantage of realizing complete coupling of network information including topology and flow. While both models can be used to simulate the dynamic behavior of real complex systems and qualitatively reveal their dynamic patterns, CMLs focus more on the topology coupling strength and passenger flow coupling strength between node characteristics than load-capacity model. Therefore, CMLs are more suitable for studies considering both topology and passenger flow coupling strength. The cascading failure of the Shanghai metro network based on CMLs was analyzed [22], and the result indicated that the metro lines with more passengers generally had a more significant impact on the network vulnerability. 

However, the bidirectional flow characteristics of the metro network are not considered. The CMLs cascading failure algorithm based on the actual case of the Nanjing metro was optimized by considering the two-way metro problem and the passenger flow redistribution [23]. In fact, for the two-way metro problem, there is a highly uncertain correlation between the in-flow and out-flow, such as passenger dynamic behavior and station layout [24]. For the passenger flow redistribution, quite a number of studies conducted simulation verification by analyzing the impact of passenger flow redistribution on metro systems under emergencies [25,26,27,28]. Additionally, the result suggests that the redistributed flow in the network will increase the pressure on other nodes and affect the load balance of network traffic.

### 2.3. Graph Neural Network

To deal with the above two problems (the two-way metro problem and the passenger flow redistribution), further research needs to accurately quantify the correlation between nodes, including the passenger flow correlation and topology correlation. Deep learning can accurately extract the relationship between data objects through learning the right representation for the data. To learn a vectorized representation containing sufficient information from complex graph data, the concept of graph neural networks (GNNs) was first proposed [29], which was further elaborated [30,31]. However, these studies are computationally expensive because of propagating the neighbor information through the recurrent neural networks (RNNs) in an iterative way until the stable state. Due to the outstanding performance of convolutional neural networks (CNNs) in the computer vision domain, convolution was introduced into GNNs [32] and gradually improved [33,34,35]. 

For a metro network, the graph-based deep learning system can explore the internal relationship between nodes (or lines) and passenger flow in different time periods to provide reference for transportation planning and management. Han [36] proposed a GCN model to capture the irregular spatiotemporal dependencies along the Shanghai metro network and successfully predicted the short-term passenger flow volume.

However, GCNs still have two major limitations. One is that it is unable to handle inductive learning, which means that the images processed by training and testing are different. The other is that it is difficult to assign different weights to different neighbors for directed graphs. Therefore, recently, more variants of spatial-based GCNs have been developed [37,38]. Among them, GATs become state of the art in many datasets (e.g., Cora, Citeseer and PubMed citation network datasets) while solving the remaining problems of GCNs. Zhang [39] has applied GCNs and GATs to the traffic flow prediction and achieved great results, which demonstrates that GAT can accurately capture correlations between graph nodes.

In this paper, we aim to train passenger flow correlation between stations in the metro system with GAT, and simulate passenger flow redistribution based on this. Based on the application of CMLs model, it can compensate for the assumption that all nodes have the same coupling relationship, and capture the complex non-stationary time dynamics and spatial dependence among metro stations.

## 3. Data and Methods

### 3.1. Data Description of Shanghai Metro

**Figure 1:**
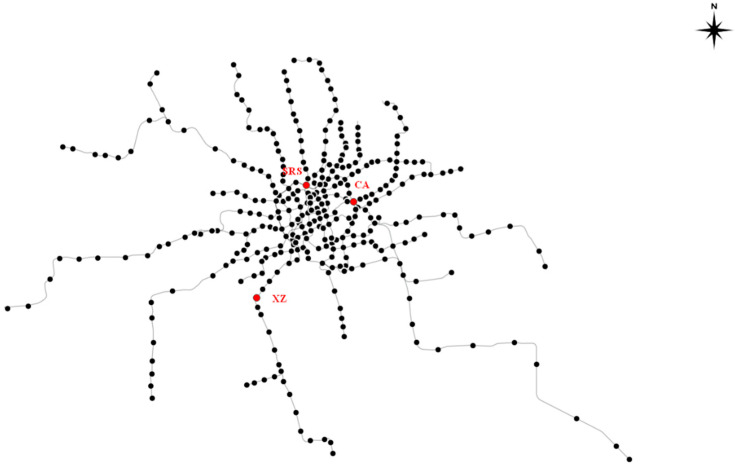
Topological network of Shanghai metro system.

As shown in Figure 1, a topological network representing the Shanghai metro system with 288 stations is built and plotted. The black dots represent the metro stations, and the links between two dots are the metro lines. In this study, an independently constructed graph data set was adopted based on the Shanghai AFC data with 288 nodes for 30 days in April, 2015. As shown in Table 1, the Shanghai AFC dataset includes the station ID, date, time, node, vehicle, cost and discount. While the AFC data can underestimate passenger demand owing to fare evasion [40], this study mainly considers the passenger flow coupling strength which was obtained by training the one-month continuous dataset through GAT. The fare evasion will have a slight effect on the training process. Moreover, since the data input in GAT’s training process is the passenger flow of one metro station every five minutes, this study does not need to consider the problem of transfer stops.

We use this data to construct the adjacency matrix of the Shanghai metro network and the graph dataset that will be learned by GAT. Moreover, RAs and IAs are used to simulate attack or failure, and the cascading failure process of the Shanghai metro system is analyzed based on the proposed improved model. Three important stations are chosen to illustrate how IAs work. They are Century Avenue (CA) with a maximum node degree of 7, Shanghai Railway Station (SRS) with maximum in-flow of 6095 and Xinzhuang Station (XZ) with maximum out-flow of 3307 in the first Wednesday of our dataset. The three chosen stations are also labelled in Figure 1. 

### 3.2. Model Specification

**Figure 2:**
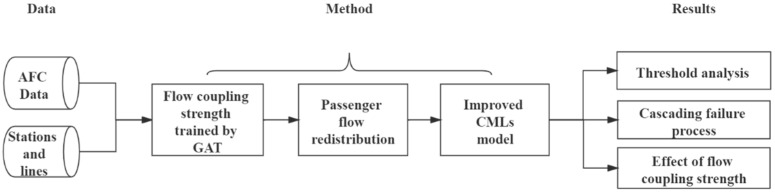
Flow diagram for cascading failure analysis of Shanghai metro system.

The research flow diagram is shown in Figure 2 and the symbols are defined in Table 2.

#### 3.2.1. Flow Coupling Strength Trained by GAT

The core of the attention mechanism is to assign weight to the given information, and information with high weight means that attention should be focused. As the sole layer throughout all the GAT architectures, a single graph attentional layer will first be described in this study. As with all attention mechanisms, a single graph attentional layer can be mainly divided into two parts: calculation of correlation coefficient and aggregation.

In our experiments, the whole metro network is a graph and each station is a graph node with operation data (e.g., passenger flow volume and speed) as its node features. The input to graph attentional layer is a set of node features, $h=\left\{{\vec{h}}_{1},{\vec{h}}_{2},\ldots ,{\vec{h}}_{N}\right\},{\vec{h}}_{i}\in {\mathbb{R}}^{F}$, where *N* is the number of nodes, and F is the number of features in each node. The output will be a new set of node features, $h\prime =\left\{{\vec{h}}_{1}^{\prime },{\vec{h}}_{2}^{\prime },\ldots ,{\vec{h}}_{N}^{\prime }\right\},{\vec{h}}_{i}^{\prime }\in {\mathbb{R}}^{F^{\prime }}$. The layer will first augment the features of each node at least by one learnable linear transformation. This step uses a shared linear transformation, parametrized by a weight matrix, $W\in {\mathbb{R}}^{F\prime \times F}$. We then concatenate the transformed features of node *i* and its neighbor *j* and map the spliced high-dimensional features by a shared attentional mechanism a to compute attention coefficients (Equation (1)):

(1) $$e_{ij}=a(W{\vec{h}}_{i},W{\vec{h}}_{j}),j\in N_{i}$$

that indicate the correlation between node *i* and node *j*. In this step, we only compute $e_{ij}$ for a pair of adjacent nodes and the shared attentional mechanism a is a single-layer feedforward neural network. To make it easier to compare attention coefficients between different pairs of nodes, we use LeakyRelu as the activation function and normalize the attention coefficients by the softmax function to get the flow coupling strengths $\varepsilon_{2}{}_{{}_{ij}}$ (Equation (2)):

(2) ε2ij=exp(LeakyRelu(eij))∑k∈Niexp(LeakyRelu(eik))

After the calculation of flow coupling strength, we aggregate the features of each node by applying a nonlinearity activation function σ (Equation (3)):

(3) $${\vec{h}}_{i}^{\prime }=\sigma \left(\sum_{j\in N_{i}}\varepsilon_{2}{}_{{}_{ij}}W{\vec{h}}_{j}\right)$$

We also employ multi-head attention to stabilize the learning process in our experiments. This step will be executed by *K* independent attention mechanisms, and then concatenate their features (Equation (4)):

(4) $${\vec{h}}_{i}^{\prime }=\overset{K}{\underset{k=1}{∥}}\sigma \left(\sum_{j\in N_{i}}\varepsilon_{{{}_{2}}_{{}_{ij}}}^{k}W^{k}{\vec{h}}_{j}\right)$$

where ∥ represents the concatenation operation, $\varepsilon_{{{}_{2}}_{{}_{ij}}}^{k}$ are the flow coupling strength computed by *k*-th attention mechanism, and $W^{k}$ is the *k*-th input shared linear transformation’s weight matrix.

**Figure 3:**
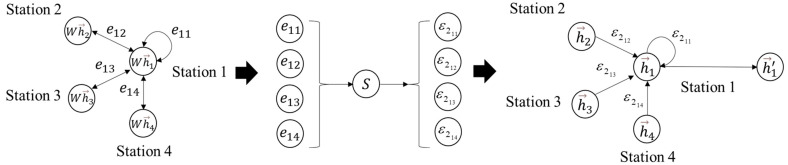
An example to describe how to obtain the passenger flow coupling strengths by GAT. W is a weight matrix for linear transformation. S is the softmax function for normalization.

Figure 3 is a simple graphical representation of GAT training coupling strength. Station 1 is the central metro station, station 2, station 3 and station 4 are the neighbor metro stations, ${\vec{h}}_{i}$ represents the characteristic information of metro station *i* (e.g., passenger flow or time). The first step is to transform the characteristic information of metro stations through a linear layer and calculate the attention coefficient $e_{ij}$ between the central metro station and adjacent metro stations. In the second step, the attention coefficient is normalized by softmax function to get the coupling strength $\varepsilon_{2}{}_{{}_{ij}}$. The third step is to generate a new feature expression by aggregating the characteristic information of the neighbor metro station and its own characteristic information to itself through coupling strength.

#### 3.2.2. Passenger Flow Redistribution

When a metro station fails because of an accident, the passenger flow of the current time step will be redistributed to other lines connected with the station according to the demand of the passenger flow itself. These redistributed passenger flows will gradually spread to the entire metro network, leading to load stress at other stations and even cascading failure. The flow coupling strength trained in Section 3.2.1 reflects the correlation of passenger flow between metro stations. We assume that this traffic correlation is still maintained between stations in the event of a failure at any station, which means that practical meaning of flow coupling strength is expressed by proportion of passenger flow redistribution. To facilitate the rapid calculation and demonstration of cascading failure process, we assume that the redistributed passenger flows are independent of each other.

If station *i* fails, the flow $p_{j\to i}$ from one neighbor of station *i* will be shared by other neighbors of station *j*. Since the link between two stations with a larger flow coupling strength means that it has stronger passenger flow carrying capacity and passenger flow preference in most cases, we assume that it will have a larger proportion of passenger flow. Then, $\Delta p_{j\to w}$ (Equation (5)) is

(5) $$\Delta p_{j\to w}=p_{j\to i}\cdot \frac{\varepsilon_{2}{}_{{}_{jw}}}{\sum_{x=1}^{k_{j}^{out}}\varepsilon_{2}{}_{{}_{jx}}}$$

where $\varepsilon_{2}{}_{{}_{jw}}$ is the flow coupling strength between station j and its neighbor station w. Equation (6)

(6) $$\sum_{w}\frac{\varepsilon_{jw}}{\sum_{x=1}^{k_{j}^{out}}\varepsilon_{jx}}=1$$

means that all passenger flow will be considered in the redistribution model.

The passenger flow redistribution model enables the relevant management agencies to obtain the transfer rules of passenger flow when a failure occurs based on the large-scale learning of the natural passenger flow movement. This indicates that the transfer of passenger flow has preference consistency in a certain period of time. Generally, this rule exists in real metro systems because when a station or line suffers a disaster or attack, passengers are more likely to choose a nearby transit service to escape chaos and accidents as quickly as possible.

#### 3.2.3. An Improved CMLs Model

CMLs have been widely used in the research of temporal–spatial traits of complex systems. Most of these researches assumed that different pairs of stations had a regular coupling topology. However, in a real metro network, the synchronization of coupling topology on CMLs does not exist due to the heterogeneity between different station pairs. Additionally, the cause of cascading failure is not only related to its topology attributes, but also related to the passenger flow transfer rules.

We first consider a CML of N nodes without heterogeneity between different station pairs and passenger flow coupling strength (Equation (7)):

(7) $$x_{i}(t+1)=R+\left|(1-\varepsilon )f\left(x_{i}(t)\right)+\frac{\varepsilon \sum_{j=1,j\neq i}^{N}a_{ij}f\left(x_{j}(t)\right)}{k_{i}}\right|$$

where $x_{i}(t)$ denotes the state of node *i* at the *t*-th time step. ε∈(0,1) is the coupling strength of topological structure. The function f defines the local dynamics of stations and is chosen in this paper as the logistic map function f(x)=4x(1−x). We use absolute value notation in Equation (7) to guarantee the nonnegative constraints of each station’s state and simulate the effect of an attack on a station by adding an external perturbation R≥1 to demonstrate that an attack or failure of the station *i* happens. A larger *R* indicates a more serious attack or failure. If node *i* has not encountered an attack, R=0.

In this work, $x_{i}(t)$ will be initially normalized in the interval (0, 1) with passenger flow at initial time of the selected dataset by the softmax function, and a larger $x_{i}(t)$ indicates heavier traffic. When 0≤x≤1, 0≤f(x)≤1. If a station *i* fails because of the passenger flow overload at time step *s*, the state $x_{i}(s)\equiv 0$ will be set for t>s. However, the neighbors of station *i* will still be affected by $x_{i}(s)$ at time *s* + 1. In case the state of one or more neighbors of the failed station is larger than threshold value 1, a new round of failures will be triggered.

If we add the passenger flow coupling strength, Equation (7) can be rewritten as Equation (8):

(8) $$x_{i}(t+1)=R+\left|\left(1-\varepsilon_{1}-\varepsilon_{2}\right)f\left(x_{i}(t)\right)+\varepsilon_{1}\sum_{j,j\neq i}\cdot \frac{a_{ji}f\left(x_{j}(t)\right)}{k_{i}}+\varepsilon_{2}\sum_{j,j\neq i}\cdot \frac{a_{ji}p_{j\to i}f\left(x_{j}(t)\right)}{q_{i}}\right|$$

where $\varepsilon_{1}$ is the coupling strength of topological structure and $\varepsilon_{2}$ is that of passenger flow, respectively, and $\varepsilon_{1},\varepsilon_{2},\varepsilon_{1}+\varepsilon_{2}\in (0,1)$. $q_{i}$ is the passenger flow of node *i*.

In order to better simulate the real metro network topological characteristics, we consider the bidirectional metro problem and improve the CMLs model with both heterogeneity between different station pairs and passenger flow coupling strength (Equation (9)):

(9) $$x_{i}(t+1)=R+\left|\begin{array}{l} \left(1-\bar{\varepsilon_{1,i}}-\bar{\varepsilon_{2,i}}\right)f\left(x_{i}(t)\right)+\sum_{j,j\neq i}\varepsilon_{1_{ji}}\cdot \frac{a_{ji}f\left(x_{j}(t)\right)}{k_{i}^{in}}+\sum_{j,j\neq i}\varepsilon_{1_{ij}}\cdot \frac{a_{ij}f\left(x_{i}(t)\right)}{k_{i}^{out}}\\ +\sum_{j,j\neq i}\varepsilon_{2_{ji}}\cdot \frac{a_{ji}p_{j\to i}f\left(x_{j}(t)\right)}{q_{i}^{in}}+\sum_{j,j\neq i}\varepsilon_{2_{ij}}\cdot \frac{a_{ij}p_{i\to j}f\left(x_{i}(t)\right)}{q_{i}^{out}} \end{array}\right|$$

We can assume $\varepsilon_{1_{ij}}=\varepsilon_{1_{ji}}$ and $\varepsilon_{2_{ij}}=\varepsilon_{2_{ji}}$ for simplicity because the in-degree and in-flow of a station are also the out-degree and out-flow of another station. Therefore, Equation (9) can be rewritten as Equation (10):

(10) $$x_{i}(t+1)=R+\left|\begin{array}{l} \left(1-\bar{\varepsilon_{1,i}}-\bar{\varepsilon_{2,i}}\right)f\left(x_{i}(t)\right)+\sum_{j,j\neq i}\frac{\varepsilon_{1_{ij}}a_{ij}}{k_{i}}\cdot \left(f\left(x_{j}(t)\right)+f\left(x_{i}(t)\right)\right)\\ +\sum_{j,j\neq i}\varepsilon_{2_{ij}}a_{ij}\cdot \left(\frac{p_{j\to i}}{q_{i}^{in}}\cdot f\left(x_{j}(t)\right)+\frac{p_{i\to j}}{q_{i}^{out}}\cdot f\left(x_{i}(t)\right)\right) \end{array}\right|$$

This formula derivation is reasonable because the out-flow of one station will not affect its state at the next time step. We use I(t) to define the cumulative failure proportion of the network before the *t*-th time step, which is the ratio of the number of station failures over the metro network with N stations. We then use V(t) to represent the instant failure proportion, which is equal to I(t) minus I(t−1). The perturbation in the station that leads to steady I(t) is defined as the critical perturbation threshold $R_{c}$, which can be used to measure the robustness of the network. A more resilient network should have more nodes with a large $R_{c}$ and a more important station to a metro system should have a larger $R_{c}$ at any time.

With this improved model, the problems needed to be studied are as follows:

- The correlation between the scale of cascading failures and the external perturbation under different attacks;
- The speed of cascading failures in a discrete time series under different attacks;
- The difference between this improved model and existing CMLs model.

## 4. Results and Discussions

### 4.1. Threshold Analysis for Cascading Failure

**Figure 4:**
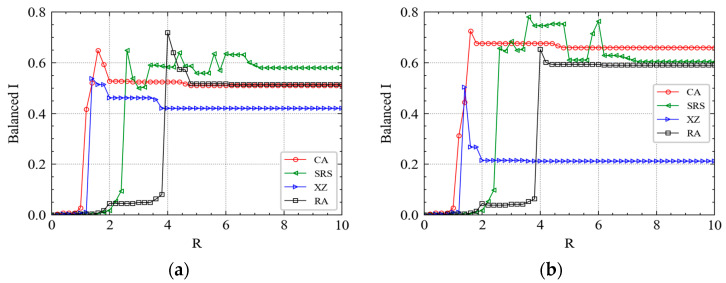

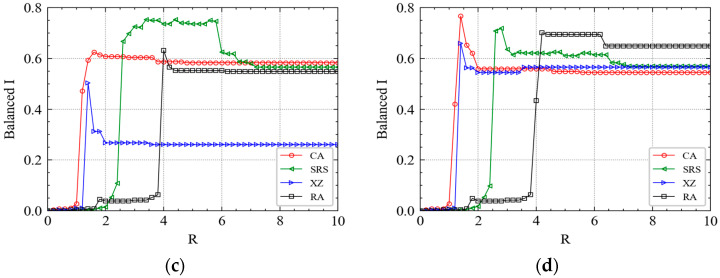
With the increase of R, the cumulative failure proportion of the system will get a balanced value under different attacks. (**a**) The morning peak of April’s first Wednesday. (**b**) The morning peak of April’s second Wednesday. (**c**) The morning peak of April’s third Wednesday. (**d**) The morning peak of April’s fourth Wednesday.

We first investigate the thresholds to trigger maximum cascading failures under different attacks. Different levels of attack have been simulated by adding different *R* to different stations. In Figure 4, we plot the variation of balanced failure proportion I with the increasing *R* in the four chosen Wednesdays of the experiment dataset. As shown in Figure 4, when R>1, failures start to be triggered in the metro topology network. However, RA and IAs obviously have different trigger thresholds. As *R* increases, the curve of CA will first make a mutation, and then that of XZ, SRS and RA. The results show that the large-scale cascading failure of IA is more easily triggered than RA because only a relatively small attack is needed to realize the rapid spread of cascading failures over the network. Thus, the Shanghai metro network is more robust to RFs than to IAs. This mutation effect also shows that for important stations, even a very small increase in the perturbation value *R* can quickly trigger large-scale cascading failure of metro stations. The results reveal the inherent vulnerability of the metro as a special mean of transportation.

From Figure 4, we observe that the stations near CA and SRS are densely distributed, while the stations near XZ are relatively dispersed. Therefore, CA and SRS have a larger flow pressure than XZ. From Figure 4,c, before the balanced *I* of XZ is held constant, there is a relatively large decrease as *R* increases. This result indicates that when the value of *R* reaches a certain threshold, the importance of CA and SRS to the whole metro network is higher than that of XZ. However, this phenomenon is not significant in Figure 4a,d. We observe that the curves have a similar trend at the first and last Wednesday of April. However, there is a significant difference in the second and third Wednesday of April. The results show that mid-month heterogeneity of stations is more significant than that at the beginning and end of the month, and the importance level has a stratified effect. It reveals the significant temporal–spatial heterogeneity of cascading failures in metro systems.

Moreover, we find that in all the subgraphs of Figure 4, the balanced *I* of each station increases firstly and then decreases instead of increasing gradually to 1. The maximum value of balanced *I* is close to 0.8, which demonstrates that after adding the topology coupling strength and passenger flow coupling strength, the Shanghai metro system is impossible to fail completely. Previous studies whose experiments guaranteed complete failure of the whole metro system as long as the perturbation *R* is large enough generally assume that $\varepsilon_{1}=\varepsilon_{2}=0.25$ [19,20,21,23]. Analyzing the mathematical formulas (e.g., Formulas (7) and (8)), the status value $x_{i}(t+1)$ of the next time step of a station is almost a fixed composition structure (when $\varepsilon_{1}=\varepsilon_{2}=0.25$): (1) 50% * the t step status value $f\left(x_{i}(t)\right)$; (2) 25% * the topology information of the neighbor node; and (3) 25% * the passenger flow information of the neighbor node.

This means that as long as the $f\left(x_{j}(t)\right)$ of the failed neighbor node is large enough, even multiplying by 0.25 will lead to the node failure. However, in Formula (10), if a station has a large number of neighbors (e.g., 7 neighbors), the $\bar{\varepsilon_{1,i}}$ and $\bar{\varepsilon_{2,i}}$ of the neighboring site can be very small (e.g., topology coupling strength = 1/7). This will lead to that the $x_{j}(t+1)$ of the neighbor node in the next time step is mostly determined by the state of the node in the current time step $x_{j}(t)$. This result shows that in the metro system loaded with real passenger flow, when the failure characteristic information is transmitted to a metro station with many adjacent stations, the state of the next time step determined by the formula is mostly borne by the state of the current time step (e.g., 80% or 90%), and the other parts jointly constitute the rest (e.g., 20% or 10%). This explains why the global failure does not occur even under very large perturbation from one initial failed station. When one station has a large number of neighbor nodes, no matter how serious a neighbor node fails, all of the neighbors share part of its next time step status information $x_{j}(t+1)$.

**Figure 5:**
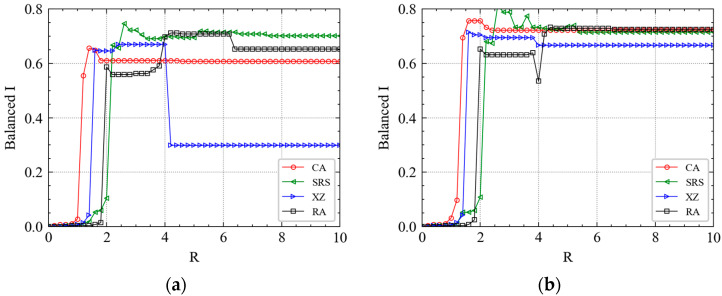

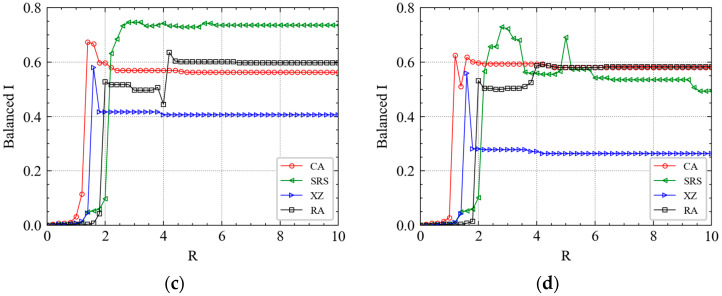
With the increase of R, the cumulative failure proportion of the system will get a balanced value under different attacks. (**a**) The evening peak of April’s first Wednesday. (**b**) The evening peak of April’s second Wednesday. (**c**) The evening peak of April’s third Wednesday. (**d**) The evening peak of April’s fourth Wednesday.

By comparing Figure 4 and Figure 5, we find that the curve of the morning peak is different from that of the evening peak. For example, the similarity of the four curves in Figure 4b,c is higher than that in Figure 4a,d. However, the similarity of the four curves in Figure 5a,d is higher than that in Figure 5b,c. It implies that for CA and SRS, the balanced *I* always increases fast when 1<R<2, then takes a little drop, finally remains constant. For XZ, the mutation rules of the morning and evening peak curves vary from time to time in a month. Most mutations occur in the interval (1, 2) and the amplitudes of them are larger. For RAs, a fast increase of cascading failure relatively requires a larger *R* than that of IAs.

The critical perturbation Rs at different time are shown in Table 3. For example, $R_{c}$ for XZ fluctuate mainly around 4, $R_{c}$ for CA fluctuate mainly around 5, $R_{c}$ for SRS fluctuate mainly around 6, and $R_{c}$s for RA fluctuate mainly around 7. The results indicate that the scale of cascading failure changes with *R*, and it also changes with time, however, the perturbation in the station that leads to steady I(t) is approximately a constant.

### 4.2. Cascading Failure Process

**Figure 6:**
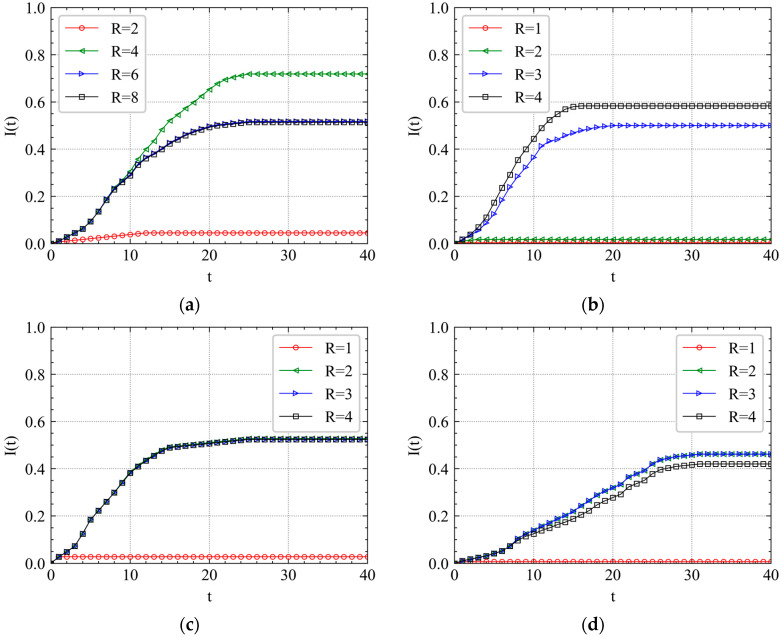
Cascading failure spreading scale analysis of April’s first Wednesday morning peak. (**a**) RA, (**b**) SRS, (**c**) CA, and (**d**) XZ.

In Figure 6, we plot the cumulative failure proportion I(t) as a function of time step *t*. When *R* is small, for example, R=1, the failure will spread in a short time and disappear quickly, and the maximum I(t) remains relatively small value, even less than 0.05. The result accords with the actual situation in which a low-level accident on a station will cause other limited metro stations to fail in a short time. It is also easier for the metro authorities to solve these problems. However, when there is a serious accident, the failure will gradually spread over the metro network. For example, in Figure 5a, when t=10, the I(t) of the curve with R=2 increases to 0.04 while that of the curve with R=4,6,8 increases to 0.3.

Compared with the curves of XZ shown in Figure 6d, the curves of SRS and CA shown in Figure 6b,c obviously trigger the maximum network failure from initial failure (t=0) more easily. SRS gets a steady value at t=15, CA gets that at t=25, while XZ gets that at t=30. Combined with the distribution of the three metro stations labeled in Figure 1, we find that the cascading failure triggered from a station surrounded by other densely distributed stations needs fewer steps to spread over the metro network.

Compared with the curves of RA shown in Figure 6a, the curves of IAs are more centralized when for larger *R*. For instance, the curves with R=3,4 in Figure 6b, the curves with R=2,3,4 in Figure 6c and the curves with R=2,3,4 in Figure 6d. Moreover, when t is small, the curves are similar with each other, especially for those with R=3,4. The result shows that it is easier for cascading failures to be predicted in the beginning and be increasingly unmanageable over time because the destruction of the metro network will restrict the passenger flow evacuation.

**Figure 7:**
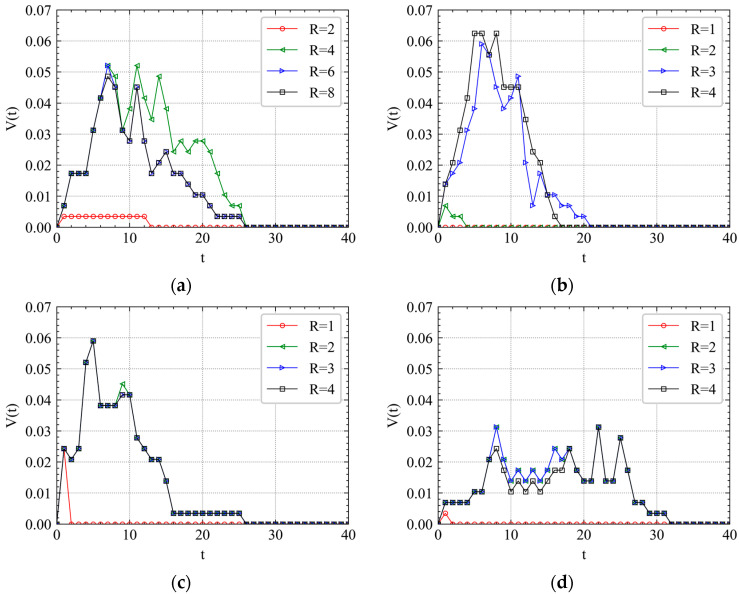
Cascading failure spreading speed analysis of April’s first Wednesday morning peak. (**a**) RA, (**b**) SRS, (**c**) CA and (**d**) XZ.

It can be seen from Figure 7 that there is an approximate normal distribution visually between instant cascading failure speed and time step *t*, which is verified by the Kolmogorov–Smirnov normal distribution test in Table 4. If *p* value >0.05, the data is normally distributed. When *R* is small, the peak times of curves under both RA and IAs are always within two steps. It indicates that the cascading failure triggered by a slight accident or attack will be limited in a small-world network and terminate quickly. However, the peak time will significantly be delayed as *R* increases to a certain value. It implies that the propagation of cascading failures is mainly reflected in the early stage. For example, t=6 in Figure 7a, t=5 in Figure 7b, t=5 in Figure 7c, and t=8 in Figure 7d.

In Figure 7c, the curves of V(t) for different *R* are almost similar with each other, which indicates that SRS has the predictability to perturbations, at least when R=2,3,4. For IAs, we can see that XZ has several peak times (t=8,18,22,25), which is different from SRS and CA shown in Figure 7b,c. We also observe that the V(t) of XZ’s peak time is almost half of that of CA and SRS. It indicates that the station location has a great influence on the cascade failure speed. For RA shown in Figure 7a, the curves have a relatively wider peak time range. The results show that different attack can significantly influence the cascading failure process. There is a diversity for the cascading failure proportion distribution and peak time as *R* changes. When attacking a random station on a metro system, the attack often needs to be more intense, which means that a larger *R* should be added. This echoes the previous conclusion.

### 4.3. Effect of Coupling Strength

#### 4.3.1. Comparison of GAT and Classical Baseline GCN

**Figure 8:**
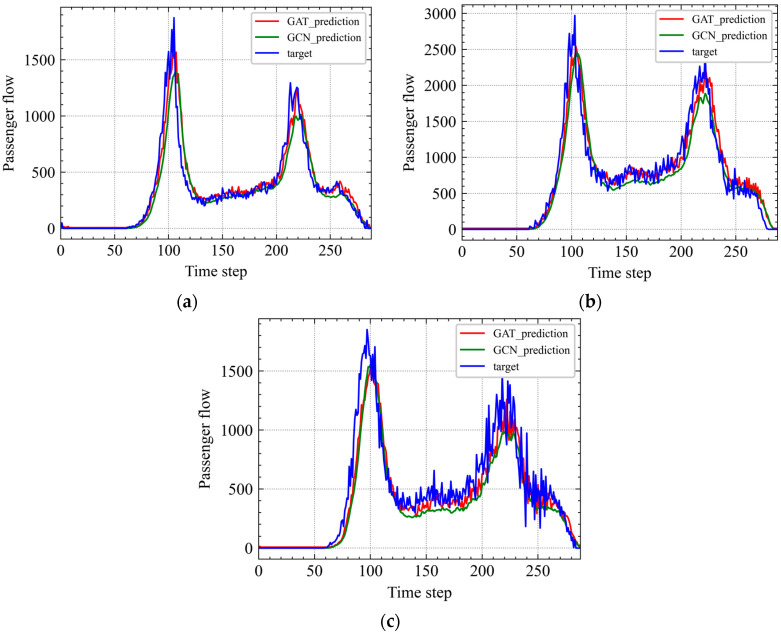
Comparison of predictions with the passenger flow target on the graph flow dataset of Shanghai metro system. (**a**) CA, (**b**) SRS, and (**c**) XZ.

As the classical algorithms of GNN, both GCN and GAT aggregate the features of neighbor nodes to the central node by learning the new node feature expression. The difference is that GCN uses the Laplacian matrix, while GAT uses the attention coefficient. In Figure 8, GCN and GAT are applied to the graph flow dataset of the Shanghai metro system to verify the superiority of GAT in learning the correlation between metro station passenger flow characteristics.

The prediction of this study is passenger flow characteristics, and the dataset includes every 5 minutes’ passenger flow. The correlation between different stations’ passenger flow can be trained by using historical passenger flow to predict the specified station passenger flow in the future, which is passenger flow coupling strength. In order to solve the problem of outlier data, this study takes the average by sliding window to make passenger flow data more stable (e.g., the first window is from 1 April 2015 to 6 April 2015, the second window is from 2 April 2015 to 7 April 2015).

Compared with GCN, GAT performs better in predicting the characteristics of the Shanghai metro passenger flow, which is shown in Figure 8 and Table 5.

#### 4.3.2. Difference between CMLs Based on GAT and CMLs

Since topology and passenger flow coupling strength describe two kinds of interactions between different pairs of stations in a network, it is necessary to consider them to investigate their influence with cascading failure. The topology coefficients and passenger flow coefficients of the chosen three stations have been shown in Table 6. XZ has two neighbors, SRS has 4 neighbors and CA has 7 neighbors. The topology coefficients of links between two stations will be equally distributed based on the number of each station’s neighbors. The passenger flow coefficients of links between two stations will be trained by GAT to get a most reasonable value. The results show that the transfer of passenger flow between each station and its neighbors is indeed unevenly distributed [23].

**Figure 9:**
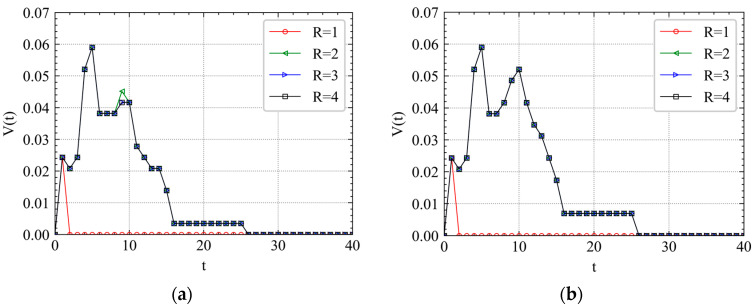
When the node with the largest degree is intentionally attacked, considering the heterogeneity between $\varepsilon_{1}$ and $\varepsilon_{2}$, the cascading failure spreading speed comparison. (**a**) CA, CMLs based on GAT, $\varepsilon_{1}\neq \varepsilon_{2}$, (**b**) CA, CMLs, $\varepsilon_{1}=\varepsilon_{2}=0.25$.

Shen [23] proposed a bi-directional CMLs model to analyze the cascading failure of Nanjing metro network. Previous studies on CMLs model assumed that $\varepsilon_{1}=\varepsilon_{2}$ [41], while this paper insists $\varepsilon_{1}\neq \varepsilon_{2}$. In this paper, a comparative experiment on Shanghai metro network is made to highlight the characteristics of CMLs based on GAT. As shown in Figure 9, for the node with the largest degree, the results have a high similarity. As CA has seven neighbors and the 7$\varepsilon_{2_{ij}}$ of CA trained by GAT are very close to each other. The result denotes that for nodes with large degree, the cascading failure process using different CMLs model can get a similar performance.

**Figure 10:**
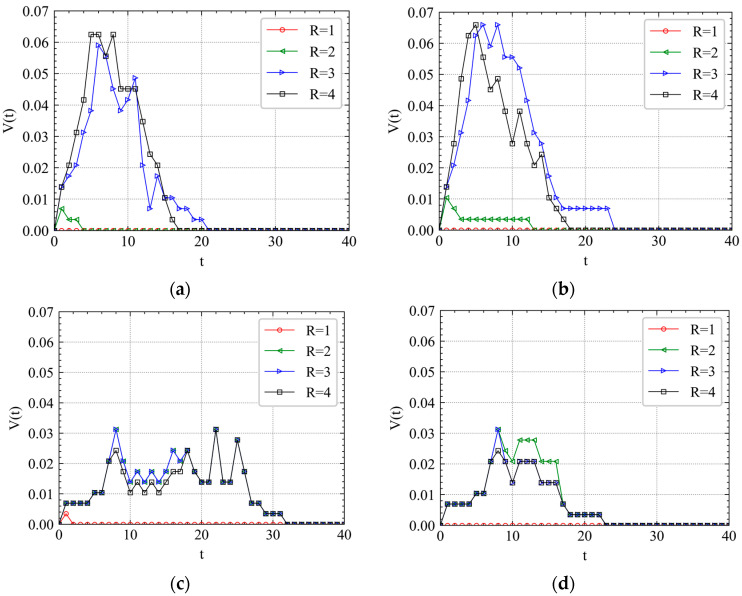
When the node with maximum in-flow (SRS) or maximum out-flow (XZ) is intentionally attacked, considering the heterogeneity between $\varepsilon_{1}$ and $\varepsilon_{2}$, the cascading failure spreading speed comparison. (**a**) SRS, CMLs based on GAT, $\varepsilon_{1}\neq \varepsilon_{2}$, (**b**) SRS, CMLs, $\varepsilon_{1}=\varepsilon_{2}=0.25$, (**c**) XZ, CMLs based on GAT, $\varepsilon_{1}\neq \varepsilon_{2}$, (**d**) XZ, CMLs, $\varepsilon_{1}=\varepsilon_{2}=0.25$.

As shown in Figure 10, for the node with maximum in-flow, $\varepsilon_{2_{ij}}\approx 0.25$, the curves R=2,3,4 of CMLs based on GAT in Figure 10a have a similar time step with that of CMLs in Figure 10b. However, for the node with maximum out-flow, $\varepsilon_{2_{ij}}\approx 0.5$, the curves R=2,3,4 of CMLs based on GAT in Figure 10c have a longer time step than that of CMLs in Figure 10d. Since the flow coupling strength in CMLs can significantly affected the propagation process of cascading failure, a more accurate flow coupling strength between stations can help to better understand the cascade failure process, which can be obtained by GAT with attention mechanism.

## 5. Conclusions

In this work, an improved CMLs model based on GAT to investigate the cascading failure process of metro networks is proposed, which provides a theoretical reference for the emergency management when the metro system encounters an accident.

By learning the real passenger flow data of the Shanghai metro network, we obtain the passenger flow coupling coefficients that can best represent the passenger flow distribution of the entire Shanghai metro network. The practical meaning of the passenger flow coupling coefficient is the proportion of passenger flow allocated from one station to adjacent stations. The dynamic OD requirements are considered to accurately simulate the network state when cascading failures occur. By analyzing the Shanghai metro topology network and passenger flow data, three key stations are selected as the targets of IAs. The simulation result of the cascading failure shows that the Shanghai metro network is more robust to Ras than to IAs. For IAs, when the degree of the perturbation increases to a threshold, the mutation effect occurs, which means large-scale cascading failure of metro stations is triggered. Previous studies insisted that a large enough perturbation or attack can cause the complete cascading failure of the whole metro network. However, in this study, the Shanghai metro system is impossible to fail completely after considering the topology coupling strength and passenger flow coupling strength, which shows the simulation is more realistic. The scale of cascading failure changes with time increasing, however, the perturbation in the station which leads to steady state is approximately a constant. Small perturbations will spread in a short time and disappear quickly, which means a low-level accident on a station will cause limited other metro stations to fail in a short time. A station surrounded by other densely distributed stations will trigger cascading failure faster. It is easier for cascading failure to be predicted in the beginning and be increasingly unmanageable over time because the destruction of the metro network will restrict the passenger flow evacuation. There is an approximately normal distribution between instant cascading failure speed and time step and the propagation of cascading failures is mainly reflected in the early stage. Different attacks can significantly influence the cascading failure process. The transfer of passenger flow between each station and its neighbors is indeed unevenly distributed. Increasing the coupling strength of one station will reduce the robustness of the whole network.

The method proposed in this paper can simulate the cascading failure in other metro systems more accurately, not only the Shanghai metro system. The simulation considers both topology attributes and passenger flow transfer rules. There are still several meaningful research aspects. For example, further research can focus on the cascading failure triggered from multiple nodes. The passenger flow redistribution algorithm should be improved by considering alternative modes of transportation. Moreover, the difference of cascading failures caused by different failure scenarios is worth analyzing.

## Figures and Tables

**Table 1:** A sample AFC data.

ID	Date	Time	Node	Vehicle	Cost	Discount
3002779092	1 April 2015	07:23:50	L3, ZhongTanRoad	metro	0.0	no
3002779092	1 April 2015	07:44:36	L4, BaoShanRoad	metro	4.0	no

**Table 2:** Symbols and definition.

Symbols	Definition
${\vec{h}}_{i}$	${\vec{h}}_{i}$ represents the characteristic information of metro station *i* (e.g., passenger flow or time).
R	R=1 means that the current metro system doubles its passenger flow.
$a_{ij}$	$a_{ij}=1$ means that there is an edge between node i and j; otherwise, $a_{ij}=0$.
$q_{i}$, $q_{i}^{in}$, $q_{i}^{out}$	$q_{i}$ is the passenger flow through node i. $q_{i}^{in}$ represents the in-flow of node i, $q_{i}^{out}$ represents the out-flow of node i.
$k_{i}$, $k_{i}^{in}$, $k_{i}^{out}$	$k_{i}$ denotes the degree of node i which is defined as the number of edges incident to node i. $k_{i}^{in}$ denotes the in-degree of station i and $k_{i}^{out}$ denotes the out-degree of station i.
$e_{ij}$	$e_{ij}$ indicates the correlation between node *i* and node *j*.
ε, $\varepsilon_{1}$, $\varepsilon_{2}$	ε∈(0,1) is the coupling strength of topological structure. $\varepsilon_{1}$ is the coupling strength of topological structure and $\varepsilon_{2}$ is that of passenger flow.
$\varepsilon_{1_{ij}}$, $\varepsilon_{2_{ij}}$	$\varepsilon_{1_{ij}}$ is the topology coupling strength of the directed edge from node i to node j, $\varepsilon_{2_{ij}}$ is the passenger flow coupling strength of the directed edge from node i to node j.
$\bar{\varepsilon_{1,i}}$, $\bar{\varepsilon_{2,i}}$	$\bar{\varepsilon_{1,i}}$ represents the topology coupling strength mean of node i and $\bar{\varepsilon_{2,i}}$ represents the topology coupling strength mean of node j.
$p_{j\to i}$	the passenger flow volume from station j to station i.
$f\left(x_{i}(t)\right)$	$x_{i}(t)$ denotes the state of node *i* at the *t*-th time step, f(x)=4x(1−x)
I(t)	I(t) defines the cumulative failure proportion of the network before the *t*-th time step.
Balanced *I*	Balanced *I* is the stable I(t) as *R* increases.
V(t)	V(t) represents the instant failure proportion.

**Table 3:** The critical perturbation threshold at different times.

$R_{c}$	Morning-1	Morning-2	Morning-3	Morning-4	Evening-1	Evening-2	Evening-3	Evening-4
XZ	4.0	3.6	3.5	3.5	4.2	4.0	4.0	4.2
CA	5.0	4.7	3.7	4.5	4.6	5.3	4.7	4.7
SRS	7.0	6.5	7.2	7.0	7.6	5.4	5.8	9.5
RA	6.0	6.2	6.2	6.3	6.3	6.5	6.5	6.3

**Table 4:** Kolmogorov–Smirnov normal distribution test.

Station	*p* Value of R=2	*p* Value of R=3	*p* Value of R=4
SRS	0.90	0.47	0.95
CA	0.82	0.84	0.84
XZ	0.59	0.59	0.58

**Table 5:** MAE, MAPE and RMSE of GAT and GCN in Shanghai metro passenger flow dataset.

Method	MAE	MAPE	RMSE
GAT	25.08	0.52	47.82
GCN	30.64	0.49	60.22

**Table 6:** Topology coupling strength and passenger flow coupling strength trained by GAT.

Station	$\varepsilon_{1_{ij}}$	$\varepsilon_{2_{ij}}$
XZ	1/2, 1/2	0.48, 0.52
SRS	1/4, 1/4, 1/4, 1/4	0.25, 0.24, 0.26, 0.25
CA	1/7, 1/7, 1/7, 1/7, 1/7, 1/7, 1/7	0.14, 0.14, 0.14, 0.14, 0.14, 0.15, 0.15

## Data Availability

The data used to support the findings of this study are available from the corresponding author upon request.
